# The information needs of children having clinical procedures in hospital: Will it hurt? Will I feel scared? What can I do to stay calm?

**DOI:** 10.1111/cch.12692

**Published:** 2019-07-18

**Authors:** Lucy Bray, Victoria Appleton, Ashley Sharpe

**Affiliations:** ^1^ Faculty of Health and Social Care Edge Hill University Ormskirk UK

**Keywords:** children, information, preparation, procedures

## Abstract

**Background:**

Children often have unmet information needs when attending hospital, and this can cause them anxiety and uncertainty. If children are prepared and informed about what will happen during a procedure, they tend to have a better experience. Finding out what children want to know before they attend hospital for procedures could provide significant benefits for children, their families, and healthcare professionals. This study set out to investigate children's perspectives of what information is important and valuable to know before attending hospital for a planned procedure.

**Methods:**

A “write and tell” activity sheet underpinned a semistructured qualitative interview with children attending hospital for a planned procedure. The interview focussed on the information children thought was important to know before a procedure. Data were analysed using content analysis techniques.

**Results:**

One hundred six children aged between 8 and 12 years old participated in the interviews. The children identified 616 pieces of information they thought would be of value to children attending hospital for procedures. These were inductively coded into three types of information: procedural, sensory, and self‐regulation. Children want to know detailed procedural and sensory information to actively construct a script of a procedure and then build on this with information about specific strategies to help them cope with and self‐regulate the situation.

**Conclusion:**

This study has identified three types of information children recognize as important in preprocedural preparation. Children construct an understanding of a planned procedure through actively scaffolding procedural, sensory, and self‐regulation information.

**Key Messages**

- Children value a scaffolded approach to gaining and building up information and understanding about a planned procedure.
- Children identify the importance of three types of information about a planned procedure: procedural, sensory, and self‐regulation information.
- Children value focussed information about individual coping strategies they can use to help self‐regulate during a procedure.
- Information provided to children before a procedure needs to be individually tailored to each child's self‐identified information needs.

## 1 INTRODUCTION

Most children will attend a healthcare setting for a procedure at some point in their childhood (Vincent & Creteur, 2017). These healthcare settings are often unfamiliar to children consisting of unfamiliar people and unknown equipment. Procedures including blood tests, radiological investigations, and physical examinations can cause children to experience anxiety as they are unsure what to expect (Carney et al., 2003; Li, Chung, Ho, & Kwok, 2016), and this can leave them feeling unprepared (Fernandes & Arriaga, 2010), anxious and frightened (Kilkelly & Donnelly, 2011), and excluded from choices and decisions relating to their healthcare (Coyne, Amory, Kiernan, & Gibson, 2014). The impact of being unprepared for procedures can cause children to experience dissatisfaction and negative feelings afterwards (Bray, Callery, & Kirk, 2012) and can also lead to a reluctance to attend hospital in the future (Duff, Gaskell, Jacobs, & Houghton, 2012).

It is generally accepted that developmentally appropriate preparatory information has a positive effect on children's experience of clinical procedures (Gordon et al., 2011; Jaaniste, Hayes, & Von Baeyer, 2007) and reduces children's anxiety (Olumide, Newton, Dunne, & Gilbert, 2009). However, children continue to have unmet information needs (Buckley & Savage, 2010; Gordon et al., 2011; Keegan et al., 2012; Lambert, Glacken, & Mccarron, 2013; Smith & Callery, 2005) and research has shown that a lack of child‐focused preparatory information can leave children reliant on their parents to relay information to them (O'Toole, Lambert, Gallagher, Shahwan, & Austin, 2016) and information relating to procedures is often written directed at parents (Bray & Sinha, 2017; Smith & Callery, 2005; Spencer & Franck, 2005; Wahl, Banerjee, Manikam, Parylo, & Lakhanpaul, 2011). This assumes that parents will understand what will happen during a procedure and know how to deliver key information to their child in a developmentally appropriate manner (Coyne, Amory, Gibson, & Kiernan, 2016). Research to date has focussed on developing and evaluating interventions to prepare and educate children for surgery (Cuzzocrea et al., 2013; Fernandes & Arriaga, 2010; Olumide et al., 2009; Tunney & Boore, 2013; Yun, Kim, & Jung, 2015), radiological investigations (Bharti, Malhi, & Khandelwal, 2016; Carter, Greer, Gray, & Ware, 2010; Szeszak et al., 2016) and procedures (Kolk, Hoof, & Dop, 2000). However, many of these interventions have been developed by adults with minimal input from children on what information would be useful to know before attending hospital for procedures.

This research study sought to understand children's perceptions of what information is important for children to know before attending hospital for a planned procedure. Our investigation was not focussed on identifying the information needs of individual children, but more generally what types of preprocedural information children consider important and would value. This investigation was part of a larger study to develop and evaluate a child‐centred app (Xploro®) to prepare children for hospital procedures.

## 2 METHODS

### 2.1 Research design and participants

The study used an exploratory qualitative child‐centred design (Kirk, 2007; Noonan, Boddy, Fairclough, & Knowles, 2016) to explore children's perspectives of information before having a planned procedure in a healthcare setting. Children were recruited from a range of clinical departments (radiology, oncology ward, outpatients, day unit) within a children's hospital in the United Kingdom at different times and on different days of the week over a 4‐month period. Clinical staff were asked to identify any children and their parents who were eligible to approach to take part. The researchers introduced themselves and gave an overview of the study. The children and their parents were then left with an information leaflet, and the researcher returned after a short while to ask if they would like to take part. In this way, an opportunistic sampling technique (Palinkas et al., 2015) was used to recruit any eligible children aged 8 and 12 years who were undergoing a procedure such as a radiological investigation (X‐rays, MRI, CT scan, ultrasound), blood test or cannulation, day surgery, oncology treatment, or medical investigation. Children were excluded if they had a moderate or severe learning disability, were under the care of psychological services for procedural anxiety, or did not have conversational English. We hoped that by recruiting children attending hospital for a procedure, they could draw on their own experiences of procedural preparation and information needs as well as thinking more broadly about the information that would be of value for children to know.

### 2.2 Data collection

Ethical approval was obtained by the author's University Research Ethics Committee and the Health Research Authority 18/SC/0023. The researchers were mindful of the anxiety some children can experience when attending hospital, so children and parents were approached to take part either before or after their planned procedure, depending on the needs of the child and the clinical service. All children provided assent to participate and their parent/carer provided written consent. Interviews were conducted in a quiet area within the clinical department.

**Figure 1:**
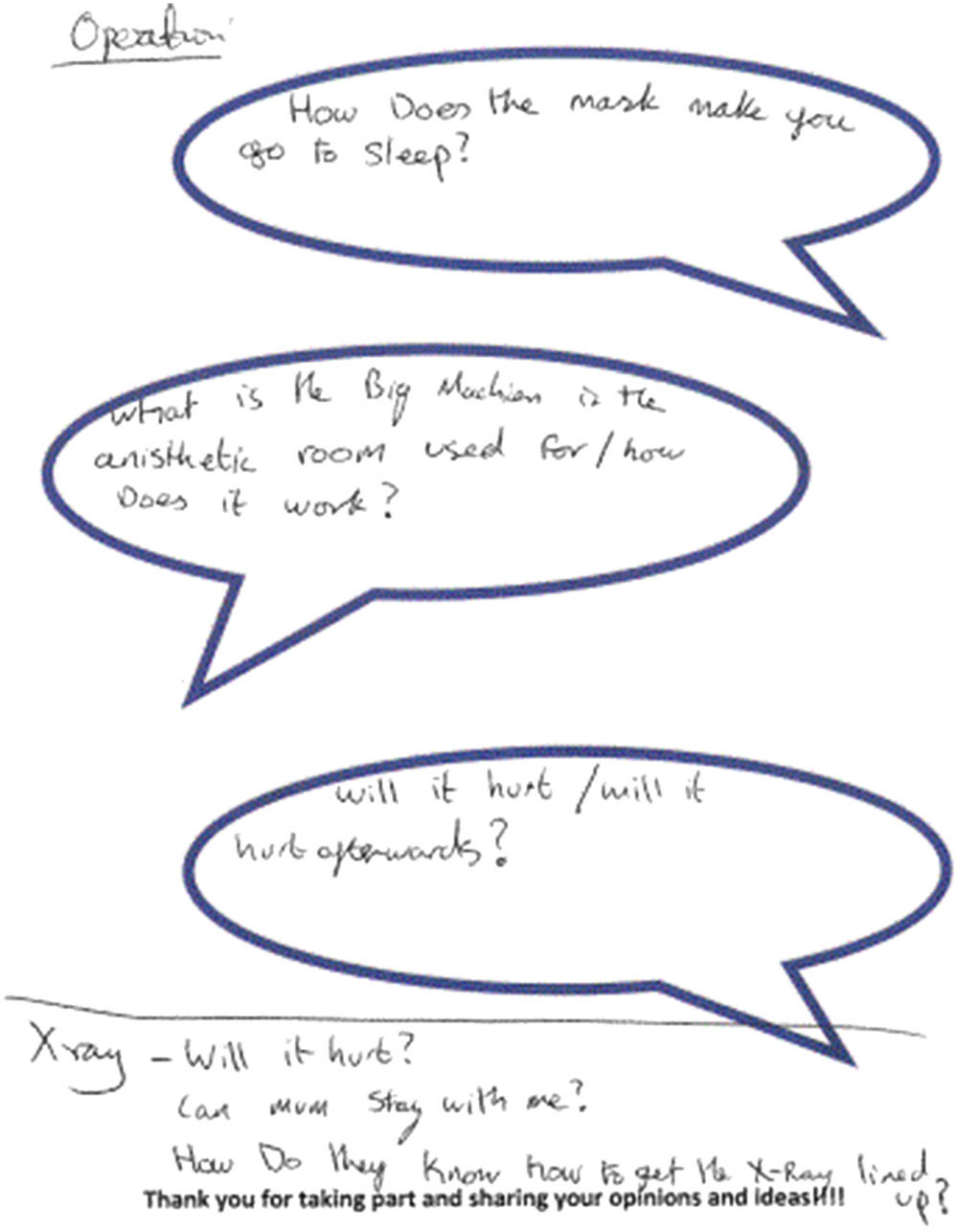
Activity sheet [Colour figure can be viewed at http://wileyonlinelibrary.com]

Semistructured interviews asked children what information they thought was important for children to know about before a planned procedure. If children were struggling to think of any information, they were prompted to think about what information they had wanted to know about before coming to the hospital for their procedure or what questions they had asked, or wished they had asked about their procedure. Children chose to either tell the researcher what they thought or write their thoughts on an activity sheet, this followed the format of “write and tell” so that information could be clarified and meanings explored (Noonan et al., 2016). The focussed activity sheets, including large speech bubbles, worked well in engaging children in the interview to share their thoughts and opinions (Figure 1). The layout and wording of the activity sheet was developed through consultation with five children and young people.

### 2.3 Analysis

The information recorded on the activity sheets was analysed using content analysis (Elo & Kyngäs, 2008), and the questions and pieces of information identified by the children were inductively coded. The codes were then refined and grouped to form categories of the types of information valued by children. The allocation of codes was carried out independently by two members of the research team; any discrepancies were discussed and consensus reached. We compared the types of information identified across the different genders, ages, and hospital experiences of the children involved in the study.

## 3 RESULTS

### 3.1 Participant characteristics

A total of 106 children (55 girls and 51 boys) with a mean age of 10.1 years participated. Seven children declined to take part. Children were attending hospital for a wide range of procedures (see Table 1) and had differing experiences of hospital with 63 of the children having attended hospital before this event.

**Table 1:** Procedure type

Treatment or procedure	Total (*n* = 106)
Radiology	26
Medical	22
Surgical	24
Blood test	20
Plaster removal	14

Six hundred and sixteen pieces of information were identified by the children as important to know about before attending hospital for a planned procedure. Some children identified over 10 pieces of information about a particular procedure and hospital visit while some only identified one or two pieces of information. The information the children identified was often based on their own experiences, questions they wished they had asked or information they had found out by chance that they would have found useful to know before their procedure. The children in this study had mainly relied on their parents as the main information provider.

Children's information needs were categorized into three main types of information: procedural information, sensory information, and self‐regulation information (see Table 2). We observed that the types of information children identified as important did not vary between different ages, genders, previous hospital experiences, or between the children who were interviewed before their procedure and those interviewed afterwards.

**Table 2:** The identified information needs of children attending hospital for a planned procedure

Type of information	Pieces of information identified (*n* = 616)	Number of children identifying this type of information	Most frequently identified information needs within each type
Procedural information	452	106	How long will the procedure take?
			What does the machine or equipment look like?
			Who are the different staff?
			How does the machine or equipment work?
			What will happen?
Sensory information	120	74	Will the procedure hurt?
			How will the procedure feel?
			How will the medicine taste?
			Will I be okay?
			Will I be scared?
			How will the procedure make me feel?
Self‐regulation information	44	34	Are parents allowed to stay with me?
			Can family visit?
			Who will be with me for the procedure?
			How can I stay calm?
			Can I have my iPad?
			Do you have to look at the screen?

**Figure 2:**
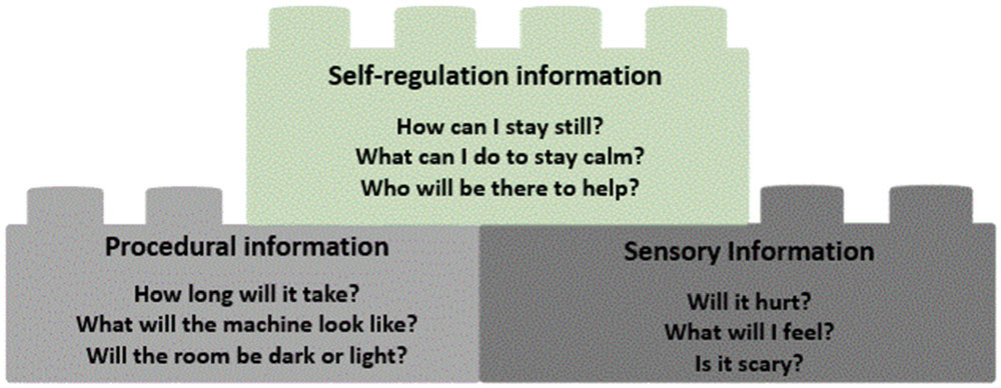
Constructing an understanding of a planned procedure [Colour figure can be viewed at http://wileyonlinelibrary.com]

All children identified that it was important to sequentially know procedural then sensory information, and this then led onto many of the children (*n* = 34) acknowledging the value of self‐regulation information in helping to be prepared for and cope during procedures. The information children identified as important preprocedure seemed to underpin a scaffolded approach to gaining information, asking questions to piece together and build up information about a planned procedure (Figure 2).

### 3.2 Procedural information: “What will happen?”

All the children identified that it was important for children to have detailed information about a procedure (452 pieces). Children identified that it was important to know information around the appearance of the procedure room such as “what will the room look like” or “will the room be dark.” Information around the context included “how many people will be there” and “who the people will be.” Some children also thought it was important for children to know very specific information about the environment such as “will there be a radiator?” They thought this would help children “picture the room before they get there.” They also highlighted that it is important for children to know about broad issues relating to a hospital visit such as what food would be available in hospital and more unusual information such as the quality of the care delivered in a hospital, for example, “what standard is the hospital?”

Every child identified the importance of information centred on knowing more about a procedure such as “what the machine does” or to know why a certain procedure was being undertaken, “why do I have to have a needle?” Children thought it was important to know about the equipment they may encounter such as “what cannulas look like” or “what drip stands are for.” Some children also thought it was important to know the trajectory of a procedure, “what will happen,” “who will you see first,” “when will it be done,” and “how long will it take.” Children also identified it was important to know who would be undertaking a procedure, for example, “why does the nurse take blood and not the doctor.” Children wanted honest information about what could go wrong during a procedure such as “can a needle go right through your arm” and “can you die?”

### 3.3 Sensory information: “Will I feel scared?”

The children identified that it was important to know information around the sensory aspects of a procedure (120 pieces), relating to what a child may experience during a procedure (touch, taste, smell, hear) and the emotions a child might feel before, during, and after a procedure.

The piece of information children identified as most important to know about a procedure was “will it hurt?” The children identified that this was important to know regardless of whether a child was visiting hospital for an invasive procedure such a blood test or for a noninvasive procedure such as an X‐ray. All the children who were having a blood test or insertion of a cannula identified that it was important for children to know whether they would experience pain. The children used words such as “hurt,” “sharp,” “pain,” “numb,” and “sore” as well as “tickle” and “feel weird” to discuss how having the test or cannula could be described to other children. The children who were having surgery mainly identified that it was most important to know about the anaesthetic and how a child may feel “going to sleep” or “waking up.” Children thought information should include details about feelings such as “what the gel will feel like” (for ultrasounds), “what the drink will taste like” (barium for a radiological investigation), “what the wipes will smell like” (the alcohol wipes used before a blood test), and “how numb feels” (local anaesthetic cream applied for cannula insertion).

It was also identified as important for children to be given information around how they may feel when undergoing a procedure, such as “will I be happy” or “will I feel scared?”

### 3.4 Self‐regulation information: “What can I do to stay calm?”

The final theme identified information around “self‐regulation” (44 pieces). Not all children identified that it was important to know self‐regulatory information; however, those with a longer hospital stay or those undergoing more invasive treatments such as surgery or blood tests all identified that it was useful to be provided with information about how to cope during a procedure. They thought it was important for children to have information about “how to stay calm” and how to self‐distract during a procedure, “can you watch an iPad?” It was also identified as important for children to know who would be with them to provide support during a procedure: “can your mum stay with you,” “who else can be with you.” It was perceived as important for children to know that they could also be supported by familiar and comforting objects such as a teddy or favourite comforter.

## 4 DISCUSSION

This study adds an understanding of the types of information valued by children when they are visiting hospital for a planned procedure. This is important for anyone involved in developing information and resources for children and also parents and health professionals who interact with children prior to planned clinical procedures. This study was not concerned with the timing or format of information provision, which is to some extent well evidenced (Jaaniste et al., 2007), but on the types or content of information children identify as important and the way children piece together or scaffold preprocedural learning. The scaffolded approach to learning (Hammond, & Gibbons, 2005; Wood & Middleton, 1975) recognizes how information is pieced together to create a whole and deeper understanding of a particular topic in order to solve a problem: in this instance, children gaining an understanding of a planned procedure in order to cope and get through it. Scaffolding also refers to how children's learning can be facilitated by adults following children's lead (Pesco & Gagné, 2017) and responding to their self‐identified information needs. There is only minimal evidence within the literature that considers how children piece together or “build up” procedural information (Jaaniste et al., 2007), with the focus being on children's broader understandings of health, treatment, or illness information (Coyne, 2006; Smith & Callery, 2005). This study has demonstrated that children are competent to identify preprocedural information needs and value being supported to actively construct this information to develop an individualized understanding of a procedure.

Children expressed that detailed preprocedural information about who will be there (actors), the environment (scene), and what will happen (the plot) helps them develop a realistic “sequential representation” or script (Eiser, 1989; Gruendel, & Nelson, 1986) of a clinical procedure. Children identified, as they have in other studies (Eiser, 1989; Jaaniste et al., 2007), that information needed to be detailed, specific, and individualized and was less helpful if it was broad or generic. This detailed procedural information seemed to provide a basic frame for children to then build on through scaffolded exploration (Darling‐Hammond et al., 2008) to gain an understanding of sensory information, for example, how a procedure may feel. This sensory information is important as procedural information alone does not have the same impact (Jaaniste et al., 2007; Tak & Van Bon, 2006), and sensory information is important in helping children experience less stress during procedures (Armfield & Heaton, 2013; Flowers & Birnie, 2015). Having both procedural and sensory types of information helps there be less discrepancy between what is expected during a procedure and what a child will experience (Cohen, 2008; Jaaniste et al., 2007).

Children's firsthand accounts from this study adds to our understanding of preprocedural “information provision” (Gordon et al 2010; Jaaniste et al., 2007) and reinforces that many children seek more than just procedural and sensory information but also value information specifically focussed on self‐regulation and coping strategies. There is some evidence to support the importance of providing children undergoing procedures with information on coping strategies (Melamed & Ridley‐Johnson, 1988), but currently, this type of information only tends to be explored with children who have experienced difficulty during previous medical procedures (Jaaniste et al., 2007). Information on self‐regulation and coping was, for many children, the final building block in constructing an understanding of a planned procedure and can be seen to enable children to rehearse, plan, and practice specific strategies to get through their procedure (Hockenberry et al., 2011). Many of the children in this study identified that knowing and having the chance to think about and rehearse strategies such as “how to sit still” or “how to stay calm” would help to develop a more meaningful or authentic script of what would happen during their procedure.

In order to build up and piece together information to develop a detailed and authentic individualized script, children need space and time to identify their own information needs. This study supports the strong evidence that children are active knowledge builders (Hirsh‐Pasek et al., 2015; Piaget, 1965), and through questioning and interaction with information sources (materials, parents, and health professionals), children can build up an understanding of what will happen during a procedure, how the procedure may be experienced, and strategies to help them cope or self‐regulate. Interaction is important to provide children with the opportunity to reinforce their understanding of information (Hirsh‐Pasek et al., 2015). This study provides a useful way to consider the types of information valued by children, but it is important to recognize that each child's information needs and circumstances differ; this study demonstrated that although there was commonality in the types of information identified as important by children, there was a wide variability in the actual pieces of information or questions children thought were useful to know. The reliance on leaflets, as an information‐giving technique (Patel, Cherla, Sanghvi, Baredes, & Eloy, 2013), without meaningful discussion, is likely to fall short of enabling children to actively construct knowledge or provide children with the sensory and self‐regulation information tools they need to develop meaningful scripts. There is a need for health professionals and information developers to acknowledge the different types of preprocedure information children value and ensure that these are addressed in a way which acknowledges children's ability to actively construct understanding and develop individualized scripts of procedures.

## 5 LIMITATIONS

This study only focussed on the information children aged 8–12 years thought was important to know before attending hospital for procedures. We recruited children who were already in hospital and were about to undergo a procedure or had already had their procedure conducted. This is likely to have influenced the information they identified as important and may be different to the information identified by children out of the hospital context.
